# Anthocyanins from *Hibiscus syriacus* L. Inhibit Oxidative Stress-Mediated Apoptosis by Activating the Nrf2/HO-1 Signaling Pathway

**DOI:** 10.3390/antiox9010042

**Published:** 2020-01-03

**Authors:** Ilandarage Menu Neelaka Molagoda, Kyoung Tae Lee, Yung Hyun Choi, Gi-Young Kim

**Affiliations:** 1 Department of Marine Life Sciences, Jeju National University, Jeju 63243, Korea; neelakagm2012@gmail.com; 2 Forest Biomaterials Research Center, National Institute of Forest Science, Jinju 52817, Korea; leekt99@korea.kr; 3 Department of Biochemistry, College of Oriental Medicine, Dong-Eui University, Busan 47227, Korea; choiyh@deu.ac.kr

**Keywords:** *Hibiscus syriacus* L., oxidative stress, reactive oxygen species, nuclear factor erythroid 2-related factor-2, heme oxygenase-1

## Abstract

*Hibiscus syriacus* L. is distributed widely throughout Eastern and Southern Asia and considered as the national flower of South Korea. The extraction of several plant parts of *H. syriacus* L. is currently used as a natural remedy for several diseases, including breast and lung cancer, microbial infection, and chronic inflammation. However, the effect of the anthocyanin extract of *H. syriacus* L. petals (PS) in oxidative stress conditions has not been studied. In this study, we evaluated the cytoprotective effect of PS against H_2_O_2_-induced oxidative stress in HaCaT keratinocytes. In this study, we found that PS significantly inhibited H_2_O_2_-induced apoptosis of HaCaT keratinocytes. We also revealed that PS mediated-cytoprotective effect was associated with the increased expression of heme oxygenase-1 (HO-1) arising from the activation of nuclear factor erythroid 2-related factor-2 (Nrf2). PS also decreased H_2_O_2_-induced excessive intracellular ROS generation and restored H_2_O_2_-induced mitochondrial depolarization through the downregulation of mitochondrial ROS production. Furthermore, H_2_O_2_-induced Bax and caspase-3 expression was markedly abolished in the presence of PS. The inhibition of HO-1 by zinc protoporphyrin significantly attenuated the cytoprotective effect of PS in H_2_O_2_-treated HaCaT keratinocytes along with ROS generation, indicating that HO-1 crucially affects PS-mediated cytoprotective properties. Collectively, our results suggested that, under H_2_O_2_-mediated oxidative stress conditions, PS sustained a normal level of mitochondrial membrane potential and ROS generation in HaCaT keratinocytes by activating the Nrf2/HO-1 axis, exerting cytoprotective effects against oxidative stress.

## 1. Introduction

Keratinocytes are the predominant cell type of the epidermis, and primarily play an important role in the formation of cellular barriers against environmental stresses such as ultraviolet (UV) radiation, heat, water loss, and chemical irritation [1]. During skin damage and infections, keratinocytes recognize damage- and pathogen-associated molecular patterns through the pattern recognition receptors, resulting in the promotion of wound healing and the transduction of danger signals [2]. Therefore, the death or damage of keratinocytes in the epidermis causes the loss of the first line immune defense system. Recently, redox balance has been shown to maintain the proper cellular and tissue homeostasis in keratinocytes through the regulation of reactive oxygen species (ROS) generation [3]. Under normal physiological conditions, ROS stimulates both wound healing and the immune defense mechanisms in keratinocytes; however, an excess of ROS promotes oxidative stress in keratinocytes, ultimately causing cellular damage and apoptosis [4]. Moreover, unmoderated oxidative stress results in undesired skin concerns, including atopic dermatitis, vitiligo, aging, and skin cancer [5,6,7,8,9]. Therefore, antioxidants help keratinocytes to maintain normal function in oxidative stress conditions by suppressing ROS generation.

Nuclear transcription factor erythroid-2-like factor (Nrf2), an evolutionary conserved leucine zipper redox sensitive transcriptional factor, is paramount for triggering the expression of antioxidant response element (ARE)-related phase 2 detoxifying genes, including heme oxygenase-1 (HO-1) [10]. Ultimately, HO-1 is the rate limiting enzyme of heme catabolism and thereby catalyzes heme to biliverdin, ferrous ion, and carbon monoxide [11]. Under normal physiological conditions, the N-terminal domain of Nrf2 is trapped by Kelch-like-ECH-associated protein 1 (Keap1) in the cytoplasm, which promotes the stabilization and ubiquitin-mediated degradation of Nrf2; whereas, once it is activated, the Neh5 domain of Nrf2 is responsible for its nuclear translocation, leading to the transactivation of HO-1 [12]. Previously, Nrf2-activating compounds such as fucoxanthin and rosmeric acid combination [13], (E)-5-oxo-1-(4-((2,4,6-trihydroxybenzylidene)amino)phenyl)pyrrolidine-3-carboxylic acid (SK-119), [14] and N-Me-trichodermamide B isolated from *Penicillium janthinellum* [15] were shown to protect keratinocytes against UV and H_2_O_2_-induced apoptosis by suppressing ROS generation, concomitant with an increase of HO-1. Overall, the Nrf2/HO-1 axis is considered as the major cytoprotective defense mechanism against ROS-induced DNA damage and apoptosis in keratinocytes.

Following oxidative stress-related death signals in keratinocytes, pro-apoptotic proteins undergo post-translational modifications, such as phosphorylation and cleavage, which subsequently release cytochrome *c* from the mitochondria in the intrinsic apoptotic pathway [16]. In this regard, B-cell lymphoma 2 (Bcl-2) family proteins are important and the balance between Bcl-2 and Bcl-2 associated protein x (Bax) ultimately determines the release of cytochrome *c* from the mitochondria [17]. Once cytochrome *c* is released into the cytosol, it interacts with apoptotic protease activating factor 1 (Apaf-1), resulting in the cleavage and activation of caspase-9, which subsequently cleaves the executioner caspases, caspase-3 and -7, to initiate apoptosis [18]. In particular, mitochondrial ROS (mtROS) stimulates the release of cytochrome *c* from the mitochondria to the cytosol by collapsing the balance of the redox systems, such as downregulation of the mitochondrial membrane potential and the oxidization of mitochondrial glutathione [19], indicating that the downregulation of mtROS protects keratinocytes from apoptosis induced by environmental insults such as UV and ROS. Recently, Kovac et al. reported that Nrf2 was involved in both cytosolic and mtROS generation via nicotinamide adenine dinucleotide phosphate oxidase [20], suggesting that Nrf2 can downregulate ROS-mediated apoptosis in the cytosol and mitochondria.

*Hibiscus syriacus* L. is the national flower in South Korea, distributed widely throughout Eastern and Southern Asia [21]. The dried root and stem bark of *H. syriacus* L. have been applied as a traditional remedy as an antidote or antipyretic. Geng et al. previously revealed that the pigment extract of *H. syriacus* L. petals possessed potential anti-oxidative properties in vitro through the upregulation of hydroxyl radical scavenging activity and the downregulation of lipid peroxidation [22], indicating that the extract can be used as a source of antioxidants. Recently, we purified and confirmed 17 anthocyanins from the flower petals of the *H. syriacus* L. variety (PS), and found that they potently inhibited melanongenesis in vitro and in vivo [23]. Nevertheless, no study has evaluated the antioxidant effects of PS in H_2_O_2_-treated keratinocytes. In the present study, we found that PS protected HaCaT keratinocytes from H_2_O_2_-mediated apoptosis by stimulating the canonical Nrf2/HO-1 axis, which inhibits cytosolic and mtROS generation.

## 2. Materials and Methods

### 2.1. Preparation of PS

PS was cultivated in the *Hibiscus* clonal archive of the Korea Forest Research Institute, Suwon, Republic of Korea (N 37°15′5.56″, E 126°57′16.11″) and identified by H.-Y. Kwon (National Institute of Forest Science, Suwon, Korea). Voucher specimens were deposited in the Korea Forest Service (NF-H8-F). PS was prepared in our previous study [23], which contains 17 anthocyanins.

### 2.2. Reagents and Antibodies

3-(4,5-Dimethylthiazol-2-yl)-2,5-diphenyltetrazolium bromide (MTT), *N*-acetylcysteine (NAC), MitoSOX Red, MitoTEMP, MitoTracker Green, and zinc protoporphyrin (ZnPP) were purchased from Sigma Aldrich (St. Louis, MO, USA). 2′,7′-Dichlorodihydrofluorescein diacetate (DCFDA) and 4′6-diamidino-2-phenylindole (DAPI) were purchased from Molecular Probes (Eugene, OR, USA). Antibodies against Nrf2 (sc-365949), Keap1 (sc-514914), PI3K (sc-1637), p-Akt (sc-271964), Akt (sc-5298), caspase-3 (sc-7272), poly (ADP-ribose) polymerase (PARP) (sc-7150), Bax (sc-7480), HO-1 (sc-10789), β-actin (sc-69879), nucleolin (sc-13057), and peroxidase labelled anti-mouse immunoglobulins (sc-16102) were observed from Santa Cruz Biotechnology (Santa Cruz, CA, USA). Antibodies against p-PI3K (PA5-17387) and p-Bcl-2 (MA5-15046) were purchased from Thermo Fisher Scientific (Waltham, MA, USA). Peroxidase-labeled anti-rabbit immunoglobulins (KO211708) was obtained from KOMA BIOTECH (Seoul, Republic of Korea). Dulbecco’s modified eagle medium (DMEM), fetal bovine serum (FBS), antibiotic mixture, and trypsin-ethylenediaminetetraacetic acid solution were purchased from WELGENE (Gyeongsan, Korea). Alexa Fluor^®^ 488 goat anti-rabbit secondary antibody was purchased from Abcam (Cambridge, MA, UK). Dako faramount aqueous mounting media was purchased from Dako (Carpinteria, CA, USA). All other chemicals were purchased from Sigma grades.

### 2.3. Cell Culture and Relative Cell Viability

Immortalized human HaCaT keratinocytes were obtained from American Type Cell Culture Collection (ATCC; Manassas, VA, USA) and maintained in DMEM containing 10% FBS and antibiotic mixture at 37 °C in a 5% CO_2_-humidfied incubator. For relative cell viability, HaCaT cells were seeded at a density 1 × 10^4^ cells/mL overnight and then the indicated concentrations of PS (0–2000 μg/mL) were pretreated for 20 h prior to treatment with 1000 µM H_2_O_2_ for 4 h. MTT solution was incubated for 4 h at 37 °C. After removing the solution, dimethyl sulfoxide was added and then absorbance was measured at 570 nm with a microplate spectrophotometer (BioTek Instruments Inc.; Winooski, VT, USA).

### 2.4. Viable Cell Count, Viability, and Dead Cell Populations

Viable cell count, viability, and dead cell populations were measured by flow cytometry. Briefly, HaCaT keratinocytes were seeded at a density of 1 × 10^4^ cells/mL overnight and treated with the indicated concentrations of PS (0–2000 μg/mL) for 20 h followed by exposure with 1000 μM H_2_O_2_ for 4 h. Then, the harvested cells were washed with ice-cold phosphate-buffered saline (PBS) and stained with Muse^®^ Count & Viability Kit (MCH100102, EMD Millipore; Billerica, MA, USA) for 5 min. Viable cell count, viability, and dead cell populations were measured by Muse^®^ Cell Analyzer (EMD Millipore).

### 2.5. Annexin V Staining for Apoptosis

Apoptotic cell populations were determined by staining annexin V. Briefly, HaCaT keratinocytes were seeded at a density of 1 × 10^4^ cell/mL and treated with PS at the indicated concentrations (0–400 μg/mL) for 20 h followed by exposer with 1000 μM H_2_O_2_ for 4 h. The cells were washed with ice-cold PBS and incubated with a Muse^®^ Annexin V & Dead Cell Kit (MCH100105, EMD Millipore) for 30 min. Apoptotic cell populations were measured by a Muse^®^ Cell Analyzer.

### 2.6. Analysis of Intracellular ROS

The oxidation-sensitive dye, DCFDA, was used to determine the formation of intracellular ROS and NAC was used as a negative control. Briefly, HaCaT keratinocytes were seeded at a density of 1 × 10^4^ cell/mL and treated with PS at the indicated concentrations (0–400 μg/mL) for 20 h followed by exposure with 1000 μM H_2_O_2_ for 4 h. The cells were washed with PBS and immediately treated with 10 μM DCFDA. Intracellular ROS generation was measured by a GloMax^®^ 96 microplate fluorometer (Promega; Madison, WI, USA). In a parallel experiment, live imaging of HaCaT keratinocytes was detected by a CELENA^®^ S digital imaging system (Logos Biosystems; Anyang, Korea). ROS^−^ and ROS^+^ cell populations were determined by flow cytometry. Briefly, HaCaT keratinocytes were incubated with Muse^®^ Oxidative Stress Kit (MCH100111, EMD Millipore) for 30 min. ROS^−^ and ROS^+^ cell populations were measured by Muse^®^ Cell Analyzer.

### 2.7. Analysis of mtROS

HaCaT keratinocytes were seeded at a density of 1 × 10^4^ cell/mL and treated with PS at the indicated concentrations (0–400 μg/mL) for 20 h followed by exposure with 1000 μM H_2_O_2_ for 4 h. The cells were washed with PBS and incubated with 2 μM MitoSOX Red in the presence or absence of MitoTEMP. mtROS generation was measured by a GloMax^®^ 96 microplate fluorometer. For the live cell imaging, HaCaT keratinocytes were stained with 0.5 μM MitoTracker Green for 30 min and counterstained with 2 μM MitoSOX Red for 10 min. The image was taken by CELENA^®^ S digital imaging system.

### 2.8. Analysis of Mitochondrial Depolarization

HaCaT keratinocytes were seeded at a density of 1 × 10^4^ cells/mL and treated with the indicated concentrations of PS (0–400 μg/mL) for 20 h followed by exposure with 1000 μM H_2_O_2_ for 4 h. The cells were washed with ice-cold PBS and incubated with Muse^®^ MitoPotential Kit (MCH100110, EMD Millipore) for 30 min. Mitochondrial membrane depolarization was measured by Muse^®^ Cell Analyzer.

### 2.9. Analysis of Caspase3/7 Activity

HaCaT keratinocytes were seeded at a density of 1 × 10^4^ cells/mL in six-well plates overnight and treated with indicated concentrations of PS (0–400 μg/mL) for 20 h followed by exposure with 1000 μM H_2_O_2_ for 4 h. Then, the harvested cells were washed with ice-cold PBS and stained with Muse^®^ Caspase-3/7 Kit (MCH100108, EMD Millipore). Briefly, the cells were incubated fluorogenic Muse^®^ Caspase-3/7 reagent for 30 min at 37 °C followed by incubation with cell death dye, 7-AAD at 37 °C for 20 min. Caspase-3/7^+^ apoptotic cell populations were measured by Muse^®^ Cell Analyzer.

### 2.10. Protein Extraction and Western Blot Analysis

HaCaT keratinocytes were seeded at 1 × 10^4^ cells/mL in six-well plates and treated with the indicated concentrations of PS (0–400 μg/mL) for 20 h followed by exposer with 1000 μM H_2_O_2_ for 4 h. Then, the cells were harvested and lysed with a radioimmuno precipitation assay lysis buffer (iNtRON Biotechnology; Seongnam, Korea). In a parallel experiment, the cells were washed with ice-cold PBS, and cytosolic and nuclear proteins were extracted using NE-PER^TM^ nuclear and cytoplasmic extraction reagents (Pierce, Rockford, IL, USA). Protein was quantified by Bio-Rad protein assay reagents (Bio-Rad; Hercules, CA, USA). An equal amount of protein was separated by a SDS-polyacrylamide gel, transferred onto an PVDF membrane (Thermo Fisher Scientific), and then immunoblotted with the indicated antibodies. Bound antibodies were detected using an enhanced chemiluminescence plus kit (Thermo Fisher Scientific). The images were taken by ImageQuant LAS 500 (GE Healthcare Bio-Sciences AB; Uppsala, Sweden). The expressional value of cytosolic proteins was normalized to the intensity level of β-actin and nuclear proteins were normalized to nucleolin.

### 2.11. Nrf2 Immunostaining

HaCaT keratinocytes (1 × 10^4^ cells/mL) were seeded on 3% gelatin-coated coverslips and allowed to attach in cover slips overnight. Then, 400 µg/mL PS was treated in the presence or absence of 1000 µM H_2_O_2_. The cells were fixed with 4% paraformaldehyde for 10 min at 37 °C, washed three times with ice-cold PBS, and permeabilized with 0.1% Triton X-100 for 10 min at room temperature followed by washing with ice cold PBS containing 0.1% tween 20 (PBST) for 5 min each. The cells were blocked with 10% donkey serum and incubated with Nrf2 antibody (1:100 in 10% donkey serum) overnight at 4 °C. After washing with ice-cold PBST, Alexa Fluor^®^ 488 secondary antibody was added and incubated for 2 h at room temperature. Then, the cells were incubated with DAPI (300 nM) for 10 min and washed three times with ice-cold PBST for 5 min to remove excessive DAPI. The coverslips were mounted onto glass slides with Dako faramount aqueous mounting media and fluorescence images were captured by a CELENA^®^ S digital imaging system.

### 2.12. Statistical Analysis

All of the Western blots were quantified by ImageJ 1.50i (National Institute of Health, Manassas, VA, USA) and then statistically analyzed by Sigma plot 12.0. All data represented the mean of at least triplicate experiments, and were expressed as ± the standard error of the median. Significant differences between groups were determined using an unpaired one-way ANOVA test with Bonferroni correction. Statistical significance was set at *** and ^###^ *p* < 0.001, ** and ^##^ *p* < 0.01, * and ^#^ *p* < 0.05. The results shown in each of the figures are representative of at least three independent experiments.

## 3. Results

### 3.1. Low Concentrations of PS Have No Cytotoxic Effect on HaCaT Keratinocytes

**Figure 1:**
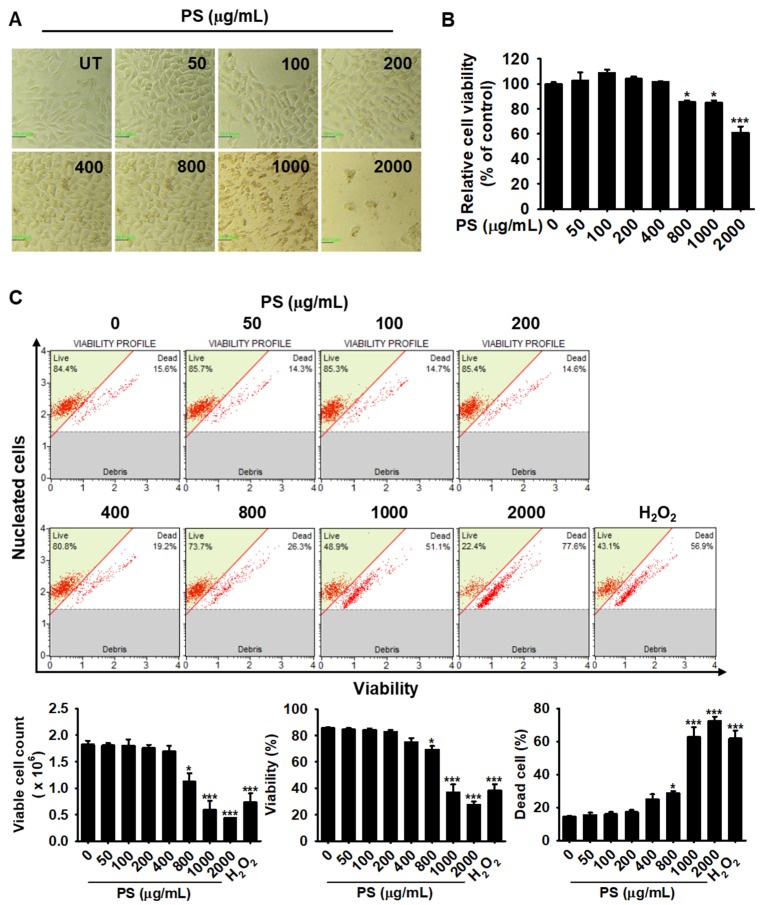
High concentrations of PS exert cytotoxic effects in HaCaT keratinocytes. HaCaT keratinocytes were seeded at a density of 1 × 10^4^ cells/mL and treated with PS at the indicated concentrations (0–2000 μg/mL) for 24 h. (**A**) Morphological changes were observed by using a phase-contrast microscope (10×); (**B**) In a parallel experiment, after treatment with PS for 24 h, MTT was added to the cells and incubated for 4 h at 37 °C, and cell viability was measured relative to the untreated control. (**C**) Under the same experimental conditions, viable cell count, viability, and dead cell populations were assessed by flow cytometry using a Muse^®^ Cell Viability Kit. H_2_O_2_ (1000 μM) was used as a positive control for the induction of apoptosis. Significant differences among the groups were determined using the one-way ANOVA followed by using Bonferroni correction. All data were averaged from three independent experiments and are presented as the mean ± the standard error of the median [*** *p* < 0.001 and * *p* < 0.05 vs. the untreated group (UT)]. Scale bars = 40 μm.

To assess the cytotoxicity of PS in HaCaT keratinocytes, we first treated cells with various concentrations of PS for 24 h. High concentrations of PS (over 800 µg/mL) gradually increased morphologically shrunk cells, and apoptotic bodies were visible when treated with 1000 and 2000 µg/mL PS; however, the damaged cells were not observed below 400 µg/mL PS (Figure 1A). Relative cell viability based on MTT activity was also decreased in a dose-dependent manner (85.4% ± 1.5%, 84.9% ± 1.9%, and 61.0% ± 4.8% by 800, 1000, and 2000 µg/mL PS, respectively); however, below 400 µg/mL PS, cell viability was similar to the untreated group (Figure 1B). As the MTT assay measures mitochondrial activity, we accurately measured the cytotoxic effects of PS treatment in HaCaT keratinocytes by using flow cytometry (Figure 1C). Consistent with the morphological changes and relative cell viability, high concentrations of PS significantly decreased the viable cell count [(1.1 ± 0.2) × 10^6^ cells/mL, (0.6 ± 0.2) × 10^6^ cells/mL, and (0.4 ± 0.3) × 10^6^ cells/mL at 800, 1000, and 2000 µg/mL PS] and proportion of viable cells (69.3% ± 2.7%, 36.9% ± 6.0%, and 27.5% ± 2.5% at 800, 1000, and 2000 µg/mL PS; Figure 1C). In addition, high concentrations of PS increased the populations of dead HaCaT keratinocytes (27.4% ± 1.2%, 62.8% ± 5.8% and 72.5% ± 2.5%, respectively, at 800, 1000, and 2000 µg/mL PS). However, below 400 µg/mL PS, viable cell count, viability, and dead cell populations remained similar to the untreated group. Therefore, low concentrations of PS (below 400 µg/mL) were selected for further study.

### 3.2. PS Protects HaCaT Keratinocytes from H_2_O_2_-Induced Apoptosis

**Figure 2:**
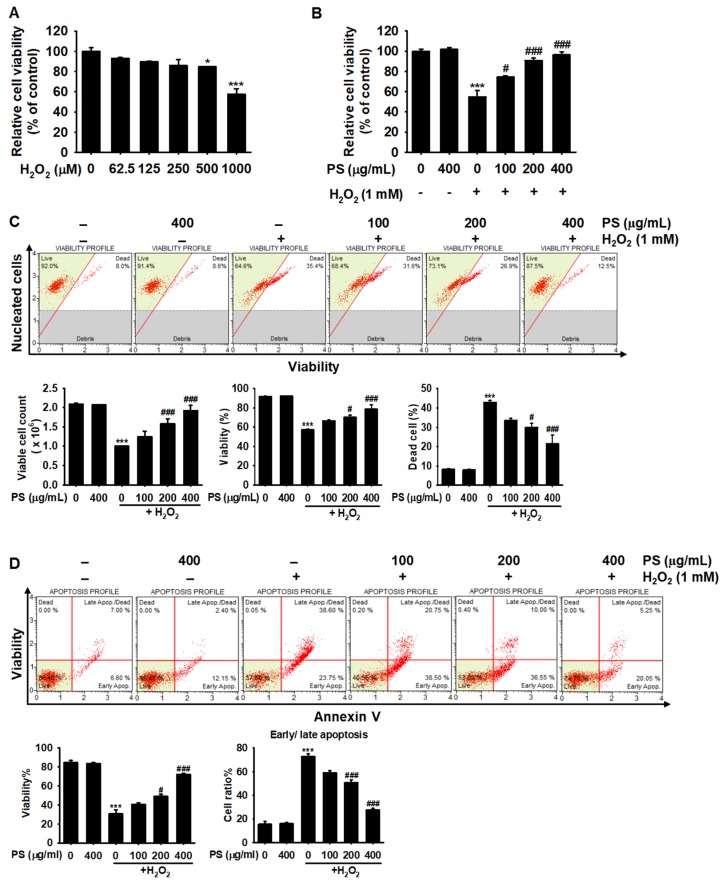
PS protects HaCaT keratinocytes against H_2_O_2_-induced apoptosis. (**A**) HaCaT keratinocytes were seeded at a density of 1 × 10^4^ cells/mL and treated with H_2_O_2_ at the indicated concentrations (0–1000 μM) for 4 h. Relative cell viability was measured by the MTT assay. (**B**–**D**) In a parallel experiment, HaCaT keratinocytes were treated with the indicated concentrations of PS (0–400 μg/mL) for 20 h prior to exposure to 1000 μM H_2_O_2_ for 4 h. (**B**) Relative cell viability was measured by the MTT assay. (**C**) Viable cell count, percentage of viability, and dead cell populations were analyzed by flow cytometry using Muse^®^ Count & Viability Kit. (**D**) Annexin-based viability and early/late apoptosis were measured by flow cytometry using Muse^®^ Annexin V & Dead Cell Kit. Graphs represent viability and proportion of cells in early/late apoptosis. Significant differences among the groups were determined using one-way ANOVA followed by Bonferroni correction. All data are averaged from three independent experiments and are presented as mean ± the standard error of the median [*** *p* < 0.001 and * *p* < 0.05 vs. the untreated group (UT) and ^###^ *p* < 0.001 and ^#^ *p* < 0.05 vs. the H_2_O_2_-treated group].

To evaluate the optimum concentration of H_2_O_2_ to act as a source of oxidative stress, various concentrations of H_2_O_2_ (0–1000 μM) were applied to HaCaT keratinocytes for 4 h and cell viability was measured. We found that 1000 μM H_2_O_2_ significantly decreased cell viability to 57.4% ± 5.8% compared with that of the untreated group (Figure 2A). In addition, we found that pretreatment with PS for 20 h restored the viability of H_2_O_2_-treated HaCaT keratinocytes in a concentration-dependent manner (74.4% ± 1.4%, 90.6% ± 2.5%, and 96.3% ± 3.1%, respectively, at 100, 200, and 400 µg/mL PS, compared with the H_2_O_2_-treated group) (Figure 2B), indicating that PS exerts cytoprotective effects against H_2_O_2_-mediated cytotoxicity. Furthermore, flow cytometry data revealed that H_2_O_2_ significantly reduced viable cell count and percentage of viable cells to (1.0 ± 0.1) × 10^6^ cells/mL and 57.0% ± 1.0% compared with the untreated group [(2.1 ± 0.1) × 10^6^ cells/mL and 91.8% ± 0.3%)] (Figure 2C). PS markedly restored the H_2_O_2_-induced decreases in the viable cell count [(1.6 ± 0.1) × 10^6^ cells/mL and (1.9 ± 0.1) × 10^6^ cells/mL at 200 and 400 µg/mL PS] and viability (70.1% ± 2.2% and 78.5% ± 4.5% at 200 and 400 µg/mL PS). PS also reduced the H_2_O_2_-induced dead cell populations (42.9% ± 0.9%) to 29.9% ± 3.8% and 21.5% ± 7.8% at 200 and 400 µg/mL. The lowest concentration of PS (100 µg/mL) also showed cytoprotective activity against H_2_O_2_-mediated oxidative stress, but the effects were not significant. To address whether H_2_O_2_-induced cell death occurred via apoptosis and if PS protected against H_2_O_2_-mediated apoptosis, HaCaT keratinocytes were stained by using Muse^®^ Annexin & Dead Cell Kit. Flow cytometry data revealed that H_2_O_2_ resulted in 72.8% ± 4.6% apoptotic cells; however, PS significantly downregulated the proportion of apoptotic cells induced by H_2_O_2_ in a concentration-dependent manner (50.9% ± 2.3% and 27.8% ± 1.1%, respectively, at 200 and 400 µg/mL PS) (Figure 2D) concomitant with an increase in viability [49.1% ± 2.1% and 72.1% ± 1.1% at 200 and 400 µg/mL PS, respectively, compared with that in the H_2_O_2_-treated group (30.5% ± 4.4%)]. Overall, these results suggest that PS is a potential inhibitor of H_2_O_2_-induced apoptosis in HaCaT keratinocytes.

### 3.3. The Cytoprotective Effect of PS against H_2_O_2_-Induced Apoptosis Is Mediated through Modulation of Apoptosis-Related Proteins in HaCaT Keratinocytes

**Figure 3:**
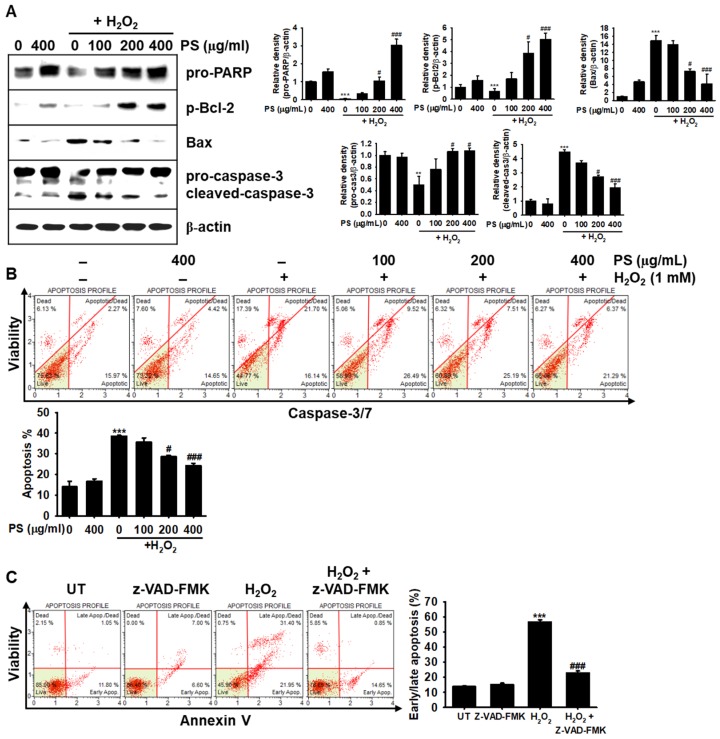
PS protects HaCaT keratinocytes against H_2_O_2_-mediated apoptosis by modulating apoptosis-related proteins. HaCaT keratinocytes were seeded at a density of 1 × 10^4^ cells/mL and pretreated with the indicated concentrations of PS (0–400 μg/mL) for 20 h prior to treatment with 1000 μM H_2_O_2_ for 4 h. (**A**) The cells were lysed with radioimmuno precipitation assay lysis buffer and Western blotting was performed to identify poly (ADP-ribose) polymerase (PARP, 116 kDa), p-Bcl-2 (26 kDa), Bax (23 kDa), and caspase-3 (32 kDa). β-Actin (43 kDa) was used as the internal control. Densitometry analysis is shown. (**B**) HaCaT keratinocytes were incubated in fluorogenic Muse^®^ Caspase-3/7 reagent for 30 min at 37 °C and then incubated with a dead cell stain, 7-AAD, at 37 °C for 20 min. Caspase-3/7^+^ apoptotic cell populations were measured by using a Muse^®^ Cell Analyzer. (**C**) In a parallel experiment, HaCaT keratinocytes were pretreated with 10 μM pan-caspase inhibitor, z-VAD-FMK, for 2 h prior to exposure to H_2_O_2_ for 4 h. Early/late apoptotic populations were then measured by flow cytometry using Muse^®^ Annexin V & Dead Cell Kit. Graphs represent caspase-3/7^+^ apoptotic cell populations (**B**) and proportion of cells in early/late apoptosis (**C**). All data are averaged from three independent experiments and presented as the mean ± the standard error of the median [*** *p* < 0.001 and ** *p* < 0.01 vs. the untreated group (UT) and ^###^ *p* < 0.001 and ^#^ *p* < 0.05 vs. the H_2_O_2_-treated group].

To evaluate whether the cytoprotective effect of PS results from anti-oxidative activity, we investigated the expression of anti-apoptotic proteins, such as PARP and Bcl-2, and pro-apoptotic proteins, such as Bax and caspase-3 in H_2_O_2_-treated HaCaT keratinocytes. As shown in Figure 3A, H_2_O_2_ resulted in a significant downregulation of PARP and p-Bcl-2; however, PS restored those protein levels in a concentration-dependent manner. In particular, 400 µg/mL PS markedly increased PARP and p-Bcl-2 levels compared with those in the H_2_O_2_-treated group, indicating that PS was a potential activator of anti-apoptotic proteins in H_2_O_2_-treated HaCaT keratinocytes. As expected, H_2_O_2_ caused a substantial induction of pro-apoptotic proteins, such as Bax, and the cleavage of caspase-3, relative to the levels in the untreated group; moreover, these effects were strongly diminished in the presence of PS, indicating that PS inhibited the expression of Bax and the cleavage of caspase-3 in H_2_O_2_-induced oxidative stress. Subsequently, it was determined whether PS downregulated caspase-3/7 activity in H_2_O_2_-mediated apoptosis because caspase-3 and caspase-7 are sequential executioners of apoptosis [18]. Flow cytometry data confirmed that PS significantly downregulated the H_2_O_2_-induced caspase-3/7^+^ population of apoptotic cells (38.6% ± 0.4%) in a concentration-dependent manner (28.6% ± 0.7% and 23.3% ± 1.2% at 200 and 400 µg/mL PS, respectively). Moreover, by using the pan-caspase inhibitor z-VAD-FMK, we confirmed the involvement of caspases in H_2_O_2_-induced oxidative stress. In the presence of z-VAD-FMK, H_2_O_2_-induced apoptosis was notably downregulated (56.7 ± 1.3% and 22.9 ± 1.4% at H_2_O_2_- and H_2_O_2_ + z-VAD-FMK-treated groups, respectively). Altogether, these data suggested that PS prevented apoptosis in HaCaT keratinocytes exposed to H_2_O_2_-mediated oxidative stress through the suppression of caspase activity.

### 3.4. PS Protects HaCaT Keratinocytes from H_2_O_2_-Induced Apoptosis by Reducing Intracellular ROS Generation

**Figure 4:**
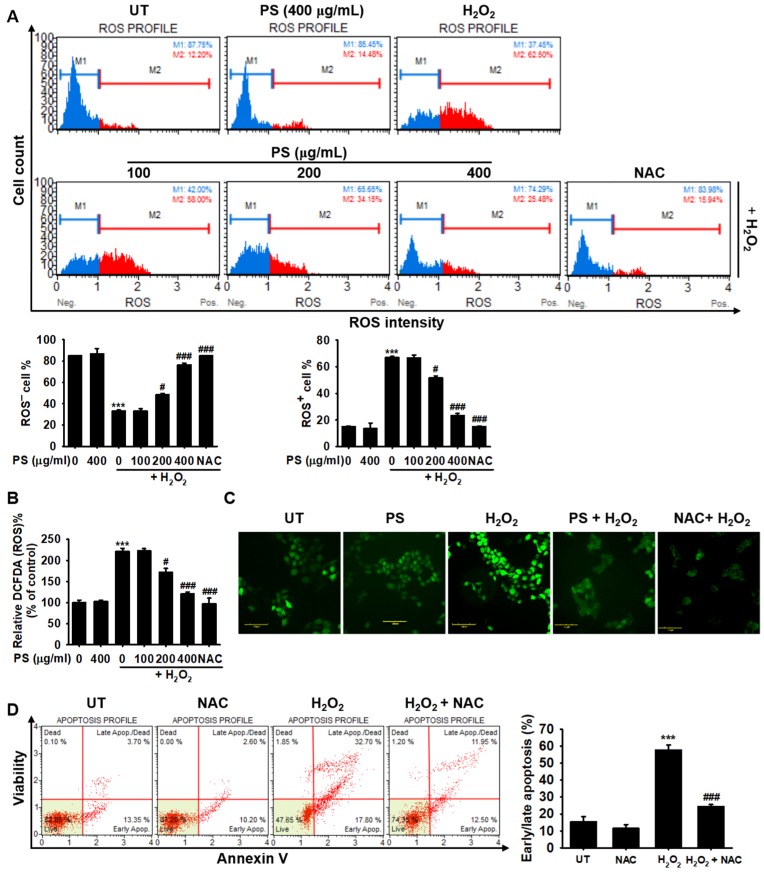
PS attenuates intracellular ROS generation in H_2_O_2_-treated HaCaT keratinocytes. HaCaT keratinocytes were seeded at a density of 1 × 10^4^ cells/mL and then stimulated with the indicated concentrations of PS (0–400 μg/mL) for 20 h prior to treatment with 1000 μM H_2_O_2_ for 4 h. *N*-Acetylcysein (NAC, 5 mM) was used as the negative control. (**A**) Intracellular ROS generation was measured by flow cytometry using a Muse^®^ Oxidative Stress Kit. Mean percentages of ROS^−^ (M1; blue) and ROS^+^ (M2; red) populations are shown. (**B**) HaCaT keratinocytes were seeded and 10 µM 2′7′-dichlorofluorescein diacetate (DCFDA) was then added to the cells. DCFDA fluorescence intensity was calculated by using a fluorometer and compared with that in the untreated group. (**C**) Live imaging stain using DCFDA was performed using a CELENA^®^S digital imaging system. (**D**) In a parallel experiment, HaCaT keratinocytes were pretreated with 5 mM NAC 2 h before treatment with H_2_O_2_ for 4 h. Then, the early/late apoptosis populations were measured by flow cytometry using Muse^®^ Annexin V & Dead Cell Kit. The early/late apoptotic population is presented as a bar graph. All data are averaged from three independent experiments and presented as mean ± the standard error of the median [*** *p* < 0.001 vs. the untreated group (UT) and ^###^ *p* < 0.001 and ^#^ *p* < 0.05 vs. the H_2_O_2_-treated group]. Scale bars = 100 μm.

We then verified whether PS attenuated H_2_O_2_-mediated apoptosis by suppressing intracellular ROS generation. Flow cytometry data showed that treatment with H_2_O_2_ resulted in a significant increase in intracellular ROS generation in HaCaT keratinocytes, to 66.9% ± 0.9% (Figure 4A). Pretreatment with PS potently reduced the H_2_O_2_-induced ROS^+^ HaCaT keratinocyte populations (51.8% ± 1.5% and 23.4% ± 1.5% at 200 and 400 µg/mL PS, respectively). Treatment with 5 mM NAC resulted in the reduction of the ROS^+^ HaCaT cell population to 15.2% ± 0.4%. The highest concentration of PS also restored the reduction of ROS^−^ HaCaT keratinocyte population induced by H_2_O_2_ to a level comparable with the untreated group. The protective effects of PS against H_2_O_2_-mediated oxidative stress were confirmed by measuring the fluorescence intensity of DCFDA using a fluorometer and fluorescence microscope. Treatment with H_2_O_2_ resulted in an increase of approximately 2-fold in DCFDA intensity, whereas the highest concentration of PS and NAC completely reduced the intensity compared with the untreated group (Figure 4B). Consistent with the above results, fluorescence microscopy data also confirmed that pretreatment with PS was associated with a low fluorescence intensity of DCFDA (Figure 4C). Finally, we tried to confirm the significance of intracellular ROS generation in oxidative stress-mediated apoptosis. Pretreatment with NAC markedly inhibited H_2_O_2_-induced apoptosis in HaCaT keratinocytes from 57.8% ± 2.9% to 24.4% ± 1.2% (Figure 2D). Altogether, these results suggested that PS downregulated H_2_O_2_-induced intracellular ROS generation in HaCaT keratinocytes, leading to the inhibition of oxidative stress-mediated apoptosis.

### 3.5. PS Protects HaCaT Keratinocytes from H_2_O_2_-Induced Mitochondrial Depolarization and mtROS Generation

**Figure 5:**
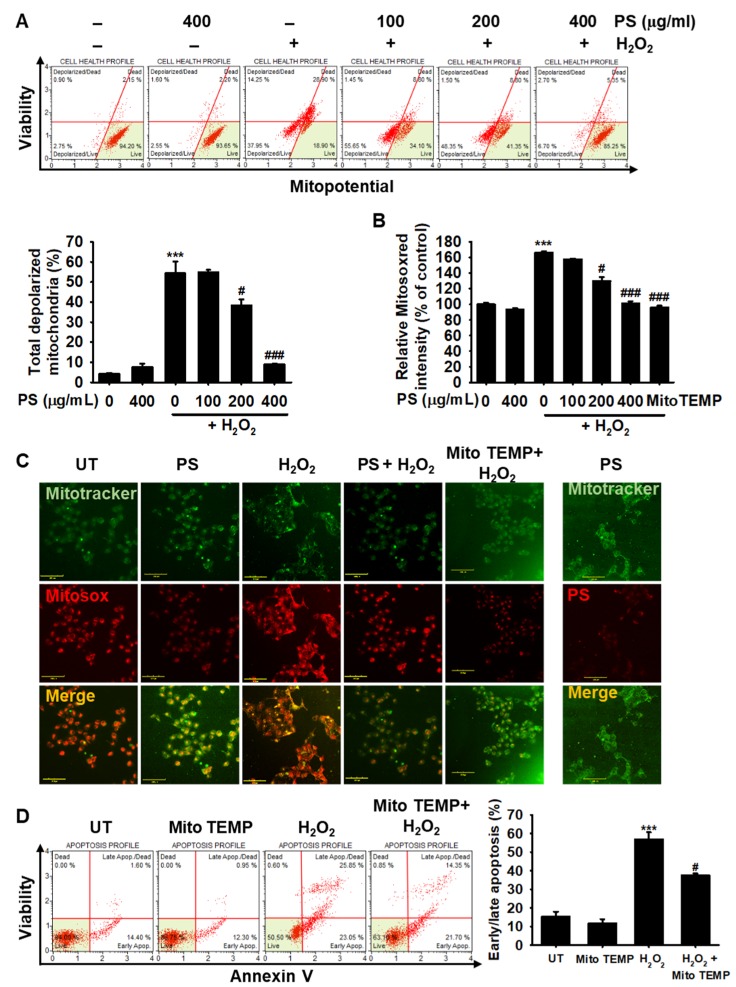
PS inhibits H_2_O_2_-induced mitochondrial membrane depolarization and mtROS-mediated apoptosis in HaCaT keratinocytes. HaCaT keratinocytes were seeded at a density of 1 × 10^4^ cells/mL and treated with the indicated concentrations of PS (0–400 μg/mL) in the presence or absence of 1000 μM H_2_O_2_ for 4 h. MitoTEMP (10 µM) was used as the negative control. (**A**) The cells were stained with Muse^®^ MitoPotential Kit and mitochondrial membrane depolarization was analyzed by flow cytometry. The mean percentages of depolarized mitochondrial populations are shown in the bar graphs. (**B**) The cells were also stained with 2 µM MitoSOX Red and the fluorescence intensity was measured by using a fluorometer. Percentage values were calculated compared to that in the untreated group. (**C**) The cells were stained with 0.5 µM MitoTracker for 30 min followed by 2 µM MitoSOX Red for 10 min. Immunofluorescence staining was performed and detected by using the CELENA^®^S digital imaging system. (**D**) In a parallel experiment, the cells were pretreated with 10 µM MitoTEMP prior to stimulation with H_2_O_2_ for 4 h. The populations of early/late apoptosis populations were measured by flow cytometry using Muse^®^ Annexin V and Dead Kit. Early/late apoptotic populations are shown in the bar graph. All data are averaged from three independent experiments and presented as mean ± the standard error of the median [*** *p* < 0.001 vs. the untreated group (UT) and ^###^ *p* < 0.001 and ^#^ *p* < 0.05 vs. the H_2_O_2_-treated group]. Scale bars = 100 µm.

Then, we investigated the effect of PS on H_2_O_2_-induced mitochondrial depolarization and mtROS generation. Treatment with H_2_O_2_ resulted in 54.4% ± 5.9% of depolarized cells, whereas pretreatment with PS decreased the percentage to 42.1% ± 2.4% and 8.8% ± 3.5% at 200 and 400 µg/mL (Figure 5A), respectively, indicating that PS stabilized mitochondrial membrane potential and blocked its oxidative stress-mediated depolarization. However, the effect was not prominent at 100 µg/mL PS (53.8% ± 1.2%) compared with the H_2_O_2_-treated group. Consistent with the mitochondrial potential data, MitoSOX Red staining revealed that treatment with H_2_O_2_ resulted in 166.6% ± 1.3% of mtROS compared with the untreated group; however, pretreatment with PS gradually decreased the intensity to 129.7% ± 5.35% and 101.2% ± 2.8% at 200 and 400 µg/mL, respectively; moreover, the effect at 400 µg/mL PS was almost comparable to that after treatment with MitoTEMP, a specific mtROS inhibitor (Figure 5B), indicating that PS downregulated H_2_O_2_-induced mtROS generation by stabilizing the mitochondrial membrane potential. Immunofluorescence staining also revealed that treatment with H_2_O_2_ significantly increased mtROS (MitoSOX Red) generation in the mitochondria (MitoTracker); however, PS completely suppressed H_2_O_2_-mediated mtROS generation (Figure 5C). MitoTEMP also significantly inhibited mtROS generation concomitant with the downregulation of H_2_O_2_-mediated apoptosis (56.9% ± 3.8% and 37% ± 1.1%, respectively, at H_2_O_2_ and H_2_O_2_ + MitoTEMP) (Figure 5D). Altogether, these results suggested that PS downregulated H_2_O_2_-mediated apoptosis by stabilizing mitochondrial membrane potential and subsequently downregulating excessive mtROS generation.

### 3.6. Under H_2_O_2_-Induced Oxidative Stress, PS Stimulates the Nrf2-Mediated Defense System in HaCaT Keratinocytes

**Figure 6:**
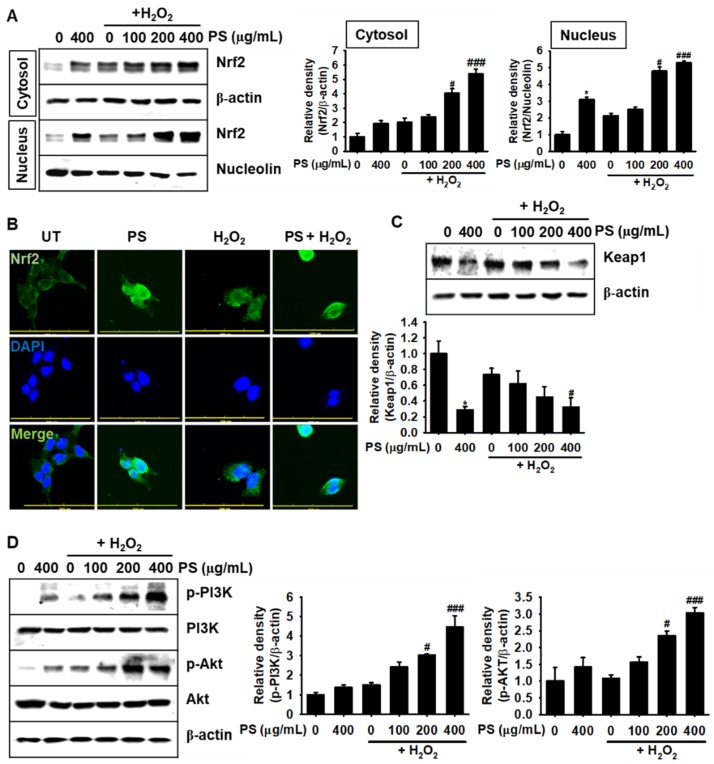
PS promotes the nuclear translocation of Nrf2 in HaCaT keratinocytes in H_2_O_2_-mediated oxidative stress. HaCaT keratinocytes were seeded at a density of 1 × 10^4^ cells/mL and treated with the indicated concentrations of PS (0–400 μg/mL) in the presence and absence of 1000 μM H_2_O_2_ for 4 h. (**A**) Western blotting was performed using cytosolic and nuclear protein fractions against Nrf2 (61 kDa). β-Actin and nucleolin (110 kDa) were used as the internal control for cytoplasmic and nuclear fractions, respectively. (**B**) HaCaT keratinocytes were cultured on 3% gelatin-coated cover slips, and the expression and nuclear translocation of Nrf2 were analyzed by immunofluorescence staining after treatment with PS (400 μg/mL) in the presence or absence of 1000 μM H_2_O_2_. In a parallel experiment, Western blotting analysis was performed to assess the expression of Keap1 (69 kDa) (**C**), and pPI3K (85 kDa) PI3K (85 kDa) and pAKT (62 kDa) and Akt (62 kDa) (**D**). β-Actin was used as the internal control along with their respective total forms. Densitometry analysis of each protein was computed by using ImageJ. All data are averaged from three independent experiments and presented as mean ± the standard error of the median [*** *p* < 0.001 and * *p* < 0.05 vs. the untreated group (UT) and ^###^ *p* < 0.001 and ^#^ *p* < 0.05 vs. the H_2_O_2_-treated group]. Scale bars = 100 μm.

As Nrf2 is a key transcription factor in oxidative stress-related defense systems [10], we investigated the possible involvement of Nrf2 in PS-mediated cytoprotection in HaCaT keratinocytes exposed to H_2_O_2_-mediated oxidative stress condition. We first prepared cytosolic and nuclear protein fractions under the indicated experimental conditions and performed Western blotting to evaluate the expression of Nrf2. Both PS and H_2_O_2_ treatment markedly upregulated the expression of Nrf2 in cytoplasm and its nuclear translocation; however, H_2_O_2_ only slightly increased the expression of Nrf2 (Figure 6A). In particular, pretreatment with PS significantly increased the nuclear Nrf2 expression, which was induced by H_2_O_2_ allowing much Nrf2 to translocate to the nucleus. The nuclear translocation of Nrf2 was further confirmed by immunostaining (Figure 6B). Treatment with PS itself markedly increased the expression of Nrf2 and promoted its nuclear translocation, which indicated that treatment with PS stimulated significant Nrf2 nuclear translocation and resulted in the initiation of anti-oxidative defense mechanisms. However, H_2_O_2_ moderately upregulated the expression of Nrf2 in the cytoplasm, which was not prominent in its nuclear translocation. In addition, Western blotting revealed that PS significantly downregulated Keap1 expression in the presence or absence of H_2_O_2_ (Figure 6C), showing that PS-mediated Keap1 degradation enabled Nrf2 to move to the nucleus. Finally, we investigated the effect PS on upstream molecules of Nrf2, including PI3K and Akt. Consistent with data on the nuclear translocation of Nrf2, PS considerably increased the phosphorylation of PI3K and Akt. These results suggested that PS activates the nuclear translocation of Nrf2 in HaCaT keratinocytes by stimulating PI3K and Akt.

### 3.7. PS-Mediated Cytoprotection Depends on the Canonical Nrf2/HO-1 Signaling Pathway

**Figure 7:**
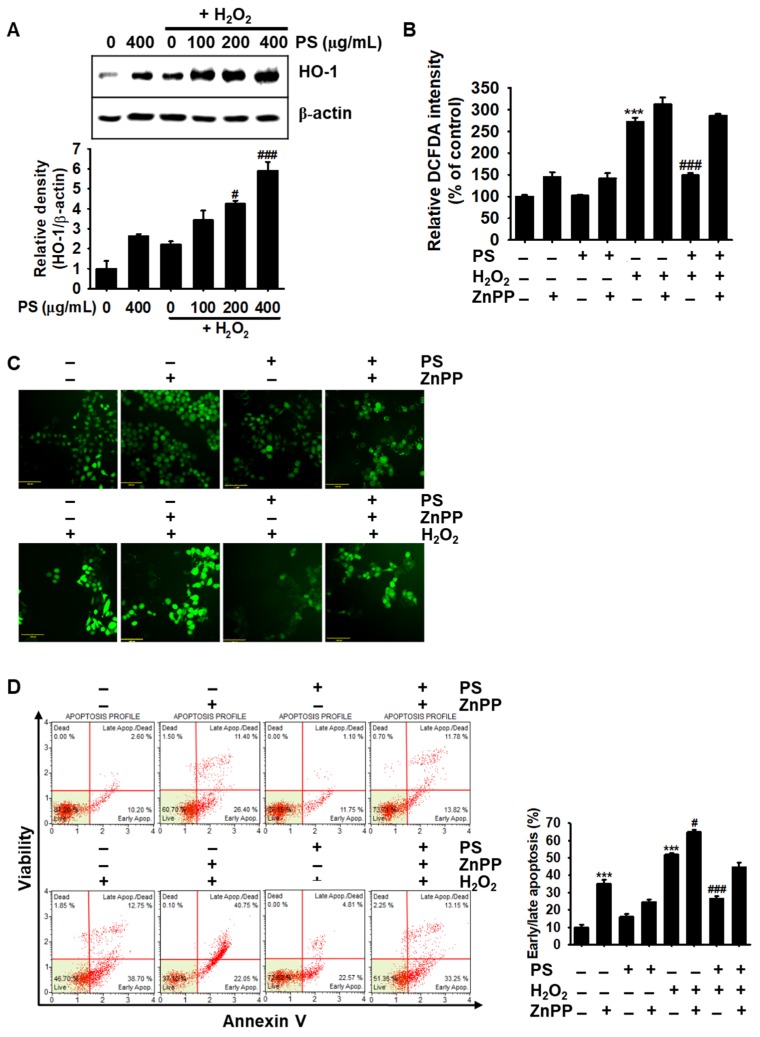
PS protects HaCaT keratinocytes from H_2_O_2_-mediated oxidative stress by activating HO-1. (**A**) HaCaT keratinocytes were seeded at a density of 1 × 10^4^ cells/mL and treated with the indicated concentrations of PS (0–400 μg/mL) for 20 h before treatment with 1000 μM H_2_O_2_ for 4 h. Western blotting was performed using total cell lysate against HO-1 (32 kDa). β-Actin was used as the internal control. (**B**) HaCaT keratinocytes were seeded and treated with 10 µM ZnPP for 2 h prior to stimulation with 400 µg/mL PS in the presence or absence of 1000 µM H_2_O_2_ for 4 h. Culture medium was replaced with 10 µM DCFDA and fluorescence intensity was detected by fluorometer. (**C**) Live cell imaging of DCFDA fluorescence was performed by using the CELENA^®^ S digital imaging system. (**D**) In the same experimental conditions, early/late apoptosis was measured by flow cytometry using Muse^®^ Annexin V & Dead Cell kit. Early/late apoptotic populations are shown in the bar graph. All data are averaged from three independent experiments and presented as mean ± the standard error of the median [*** *p* < 0.001 vs. the untreated group (UT) and ^###^ *p* < 0.001 and ^#^ *p* < 0.05 vs. the H_2_O_2_-treated group]. Scale bars = 100 μm.

As activation and nuclear translocation of Nrf2 by Akt/PI3K stimulate the expression of HO-1, which protects cells against oxidative stress and diverse toxins [12,24], we investigated if PS upregulated the expression of HO-1. We found that treatment with PS resulted in a significant increase in HO-1 expression in a concentration-dependent manner, compared with that in the H_2_O_2_-treated group (Figure 7A). The significance of HO-1 in PS-mediated cytoprotection was elaborated using ZnPP, a well-known HO-1 inhibitor. As shown in Figure 7B, the presence of ZnPP completely abolished PS-mediated inhibition of DCFDA intensity in H_2_O_2_-induced oxidative stress and considerably elevated the intensity regardless of the existence of PS, which indicates that PS inhibits ROS generation by activating HO-1 expression. In addition, treatment with ZnPP associated with the higher fluorescence intensity than that in the PS-treated group under H_2_O_2_-stimulated conditions (Figure 7C). Significance of HO-1 in H_2_O_2_-induced apoptosis were also confirmed in HaCaT keratinocytes. As shown in Figure 7D, treatment with ZnPP significantly increased apoptotic population (35.2% ± 2.2%) compared with that in the untreated group (9.9% ± 1.6%) and in H_2_O_2_-treated conditions (51.9% ± 0.7%), ZnPP further increased the populations (64.9% ± 1.2%). PS downregulated the percentage of ZnPP- and H_2_O_2_-mediated apoptosis to 24.6% ± 1.3% and 26.5% ± 1.6%, respectively; however, PS slightly downregulated H_2_O_2_-mediated apoptosis in the presence of ZnPP (44.7% ± 2.7%), indicating that HO-1 acted as the key detoxifying enzyme against H_2_O_2_-induced oxidative stress conditions. Altogether, these data suggested that PS exerts its cytoprotective effect by stimulating the canonical Nrf2/HO-1 axis.

## 4. Discussion

Keratinocytes are comprised of 90% cells in the outermost epidermal skin and produce keratins to prevent against UV radiation and preserve against pathogen infection [1,2]. In particular, UV radiation causes the activation of several signaling pathways and exacerbates DNA damage by increasing ROS generation, which triggers apoptosis of keratinocytes in the epidermis [25]. Hence, many antioxidant compounds have been reported to protect epidermal keratinocytes against oxidative stress-mediated apoptosis [26], indicating that antioxidants increase keratinocyte survival in response to adverse oxidative stress and help to treat skin damage.

The pigments from the petals of *H. syriacus* L. have been used as a food colorant, medicinal food source, and pharmaceutical compound. However, most of the biological functions of *H. syriacus* L. are not clearly understood. Recently, we revealed that PS stimulated the ERK signaling pathway, which suppresses melanogenesis [23]. Geng et al. discovered the in vitro antioxidant properties of the pigments on the inhibition of hydroxyl radical generation and lipid peroxidation [22]; however, it was not determined if PS regulated oxidative stress at the cellular level. In this study, we examined the antioxidant activities of PS against H_2_O_2_-induced oxidative stress in HaCaT keratinocytes. We found that PS was a potential candidate to protect HaCaT keratinocytes from H_2_O_2_-induced oxidative stress by activating the Nrf2/HO-1 signaling pathway.

Exogenous H_2_O_2_ potentially stimulates intracellular ROS generation by mimicking endogenous the ROS signaling pathway. H_2_O_2_ is highly diffusible through the plasma membrane and acts as a second messenger such as dibutyryl-cyclic AMP, in order to initiate signaling cascades [27]. Initiation of signaling cascades by H_2_O_2_ ultimately leads to the formation and accumulation of ROS, and thereby inhibits cell proliferation and promotes mitochondrial dysfunction-mediated apoptosis [28]. Under normal physiological conditions, mitochondrial membrane permeability is strictly regulated; however, H_2_O_2_ causes cellular apoptosis by increasing mtROS generation concomitant with the disruption of mitochondrial membrane potential, which promotes cytochrome *c* release from mitochondria to the cytoplasm by activating apoptotic Bax and inactivating anti-apoptotic Bcl-2 [29]. Released cytochrome *c* then activates caspase-9, and, in turn, stimulates effector caspases, such as caspase-3 and -7, which cleave the predominant DNA repair enzyme, PARP [30]. In this study, we found that PS protects HaCaT keratinocytes against H_2_O_2_-induced apoptosis by inhibiting mtROS generation concomitant with the inhibition of caspase-3/7, which indicates that PS is a potent antioxidant and protects epidermal keratinocytes against oxidative stress. In addition, PS restored the H_2_O_2_-induced depolarization of mitochondrial membrane potential and downregulated the Bax/Bcl-2 ratio.

Nrf2 is a redox sensitive transcription factor that regulates the transcription of phase 2 detoxifying enzymes such as HO-1, in oxidative stress conditions. Accumulated evidence suggests that Nrf2 is critical in defense systems against oxidative stress-mediated cellular damage [31]. Previously, small interference RNA (siRNA)-mediated gene silencing and knockout of *Nrf2* increased the sensitivity to H_2_O_2_-induced cellular toxicity by downregulating the expression of HO-1 [32], which indicated that Nrf2-mediated HO-1 attenuated ROS generation and consequently prevented oxidative stress-mediated apoptosis. In particular, under oxidative stress conditions, mtROS promoted cytochrome *c* release from the mitochondria, along with dysfunction of mitochondrial membrane potential, and consequently increased cellular apoptosis by activating the intrinsic pathway mediated by caspase-9 [19]. However, research performed in the past few years has suggested that Nrf2 activation may not be beneficial in certain circumstances, including some types and stages of cancer, as Nrf2 activation can promote cancer cell survival [33,34]. Nevertheless, in this study, we revealed that PS enhances the expression of nuclear translocation of Nrf2 concomitant with the expression of HO-1 and thereby protects HaCaT keratinocytes from H_2_O_2_-induced apoptosis by preserving mitochondrial membrane potential and inhibiting mtROS generation. Moreover, treatment with a specific HO-1 inhibitor, ZnPP, reversed the cytoprotective effect of PS under H_2_O_2_-induced oxidative stress, indicating that Nrf2-mediated HO-1 activation is an important route for the cytoprotective activity of PS in H_2_O_2_-treated HaCaT keratinocytes. In addition, Nrf2-deficient mice (*Nrf2^-/-^*) are associated with higher sensitivity to carcinogenesis [35] and are linked with the enhanced metastasis of cancer cells [36], which indicated that Nrf2/HO-1 also attenuated carcinogenesis and the metastasis of cancer cells. Thus, boosting Nrf2/HO-1 activity is a promising target for the treatment of inflammation and cancer.

## 5. Conclusions

In this study, we revealed that PS prevents H_2_O_2_-induced apoptosis in HaCaT keratinocytes by stimulating the canonical Nrf2/HO-1 axis. Furthermore, PS stabilized the mitochondrial membrane potential and prevented the release of cytochrome *c*. Therefore, we suggested that PS ameliorated oxidative stress-related skin damage induced by UV radiation, xenobiotics, and inflammation. However, further studies are needed to confirm the effect of PS in vivo, such as in zebrafish and/or mouse models.
